# The success of tubularized-incised plate urethroplasty in adults and children

**DOI:** 10.1186/s40064-016-2316-0

**Published:** 2016-05-27

**Authors:** Haci Polat, Umut Gulacti, Alper Gok, Mehmet Ozgur Yucel, Ali Cift, Ugur Lok, Can Benlioglu

**Affiliations:** Department of Urology, Faculty of Medicine, Adiyaman University, Adiyaman, Turkey; Department of Emergency, Faculty of Medicine, Adiyaman University, Adiyaman, Turkey

**Keywords:** Adult, Child, Hypospadias, Reconstructive surgical procedures, Urethra

## Abstract

**Purpose:**

Hypospadias repair is rarely performed in adults. It is believed that the success rate is lower in adulthood. We aimed to compare the success rate of primary hypospadias repair with tubularized-incised plate (TIP) urethroplasty in adults and children.

**Patients and methods:**

The databases of consecutive boys and adults who were treated with TIP urethroplasty for primary hypospadias between 2012 and 2015 were evaluated. All operations in the boys and adult patients were performed by a single surgeon. We considered urethroplasty complications to include a urethrocutaneous fistula, neourethral stricture, meatal stenosis, diverticulum, and glans dehiscence. Urine flow was also evaluated using uroflowmetry.

**Results:**

Seventy-seven consecutive patients underwent surgery by a single surgeon in the last 3 years for hypospadias repair. Nineteen of these patients were adults. Urethrocutaneous fistulae developed in 2 of the 19 (10.5 %) adults, and 3 of the 58 (5.2 %) boys. In addition, there were urinary tract infections in 2 (3.4 %) children, meatal stenosis in 1 (1.7 %) child, and glans dehiscence in 1 (5.3 %) adult. Uroflowmetry was normal in all patients. There was no difference in outcomes between boys and adults.

**Conclusion:**

Our data showed that the success rate of hypospadias repair with TIP urethroplasty is similar in adults and children. TIP urethroplasty is associated with good results in adults and boys.

## Background

Hypospadias repair is usually performed during preschool age. It is rarely performed in adults in developed countries. Hypospadias repair is widely believed to have a greater complication rate in adults than in children (Snodgrass et al. 2014). However, to our knowledge, there are only two studies in the literature that compare the success of hypospadias repair in adults and children. In the study of Snodgrass et al. with a small series of adult patients (six adult patients) who underwent primary distal tubularized-incised plate (TIP) urethroplasty, complication rates were similar to those reported in children (Snodgrass et al. 2014). In another study, Hensle reported that hypospadias repair in adults, performed using the same techniques as in children, had a higher complication rate (Hensle 2013); however, various flaps or 1-stage tubularized grafts were used as opposed to TIP urethroplasty.

We have performed TIP urethroplasty for primary hypospadias repair in 77 consecutive patients over the last 3 years. Nineteen of these patients were adults. We evaluated the success rate and complications of TIP urethroplasty in adult and pediatric patients in the largest adult patient series to date.

## Patients and methods

We retrospectively evaluated the medical records of consecutive boys and adults treated with primary hypospadias repair for distal or mid-penile hypospadias from 2012 to 2015. One surgeon (HP) performed the operation for all boys and adult patients. The records of patients were entered into databases including patient age, pubertal stage, meatal location, existence of penile chordee, glansplasty suture used, and urethroplasty complications, such as urethrocutaneous fistula, meatal stenosis, neourethral stricture, glans dehiscence, and diverticulum.

TIP urethroplasty was performed in all patients, by which the neourethra was translocated to the tip of the glans to create an orthotopic neomeatus. For the repair, 4/0 or 5/0 polyglactin sutures were used in children and 3/0 or 4/0 polyglactin sutures were used in adults. Patients were catheterized with a latex Foley catheter and were discharged after its removal on the second or third postoperative day.

For this study, glans dehiscence was defined as “complete separation of the glans wings.” Meatal stenosis was defined as “meatal calibration <8 Fr in symptomatic boys and <12 Fr in symptomatic men with stranguria, prolonged voiding, and/or urinary retention.” Diverticulum was defined as “visible sacculation of the urethra during voiding and/or urethrogram.”

Patients who were Tanner stages 4 and 5 were defined as adults. Only patients who underwent primary repair at our facility were enrolled in the study. Patients with <6 months of follow-up were excluded from the study.

Patients were contacted and invited back to our clinic for follow-up after an average of 15 postoperative months (range 6–38 months). These patients underwent free uroflowmetry using a digital uroflowmeter (Solar Silver, Medical Measurement System, The Netherlands); urine flow was observed, and a picture was taken at that time.

Data analysis was performed using SPSS for Windows, version 11.5 (SPSS Inc., Chicago, IL, USA). Continuous data are presented as mean ± standard deviation (SD) and/or median (minimum to maximum). Differences in continuous variables between the two groups were determined by a Mann–Whitney U test. Categorical variables were summarized as numbers and percentages and compared with the Chi square or Fisher’s exact test. A p value of <0.05 was considered statistically significant.

## Results

**Fig. 1:**
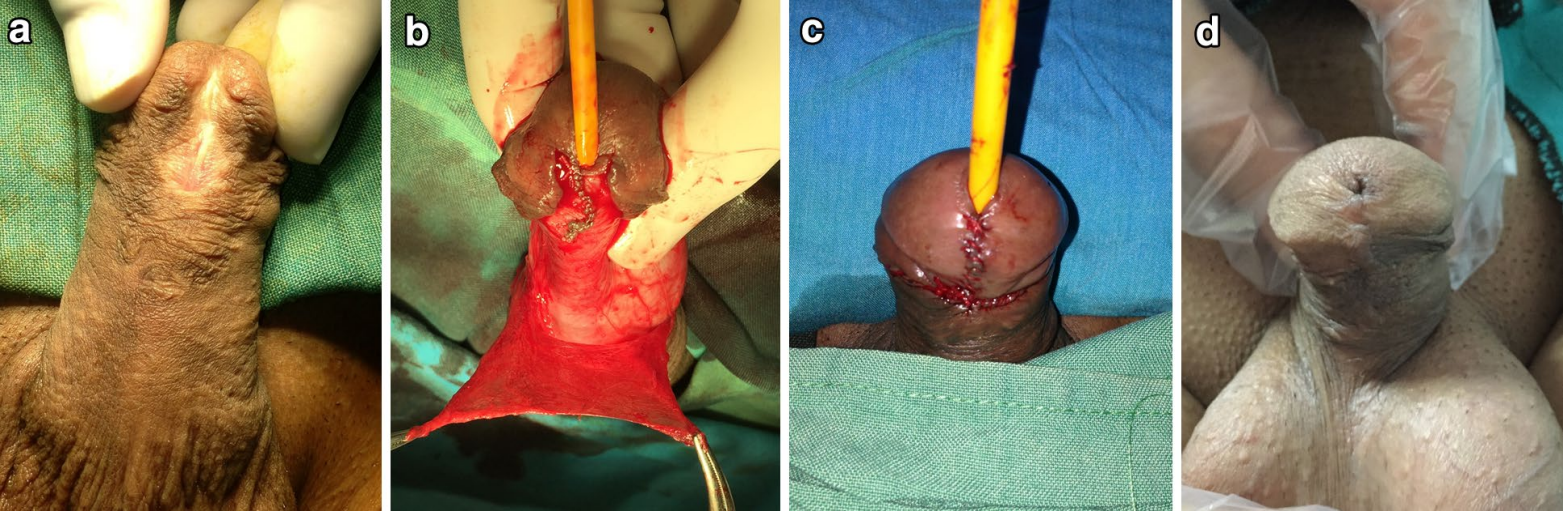
**a**–**d** A 35-year-old patient’s preoperative, intraoperative, and postoperative images

We performed 81 consecutive primary hypospadias repairs using TIP urethroplasty over the last 3 years. Seventy-seven patients with a regular follow-up were enrolled in the study. The demographic characteristics of the patients are listed in Table 1. Fifty-eight children with a median age of 2 years (mean ± SD 3.45 ± 2.939; range 1–14 years) and 19 adults with a median age of 20 (mean ± SD 23.74 ± 9.786; range 16–54 years) underwent surgery. In all patients, primary TIP urethroplasty was performed, and a neomeatus was created in the normal location at the tip of the glans (Fig. 1). Five (8.6 %) children and two (10.5 %) adult patients with mid-penile hypospadias had mild penile chordae. After penile degloving, it was determined that the clinically significant curvature did not require additional corrective surgery.

**Table 1:** Demographic characteristics of patients

	Children	Adults	*p* value
Number of patients	58	19	
Age, years			
Mean ± SD	3.45 ± 2.939	23.74 ± 9.786	<0.001
Median (min–max)	2.00 (1–14)	20.00 (16–54)	
Meatal location, n (%)			0.483
Distal	41 (70.7)	15 (78.9)	
Mid-shaft	17 (29.3)	4 (21.1)	
Existence of chordae, n (%)	5 (8.6)	2 (10.5)	1.000
Follow-up period, months			
Mean ± SD	16.76 ± 9.312	16.58 ± 9.495	0.957
Median (min–max)	14.00 (6–38)	14.00 (6–38)	

*SD* standard deviation

**Fig. 2:**
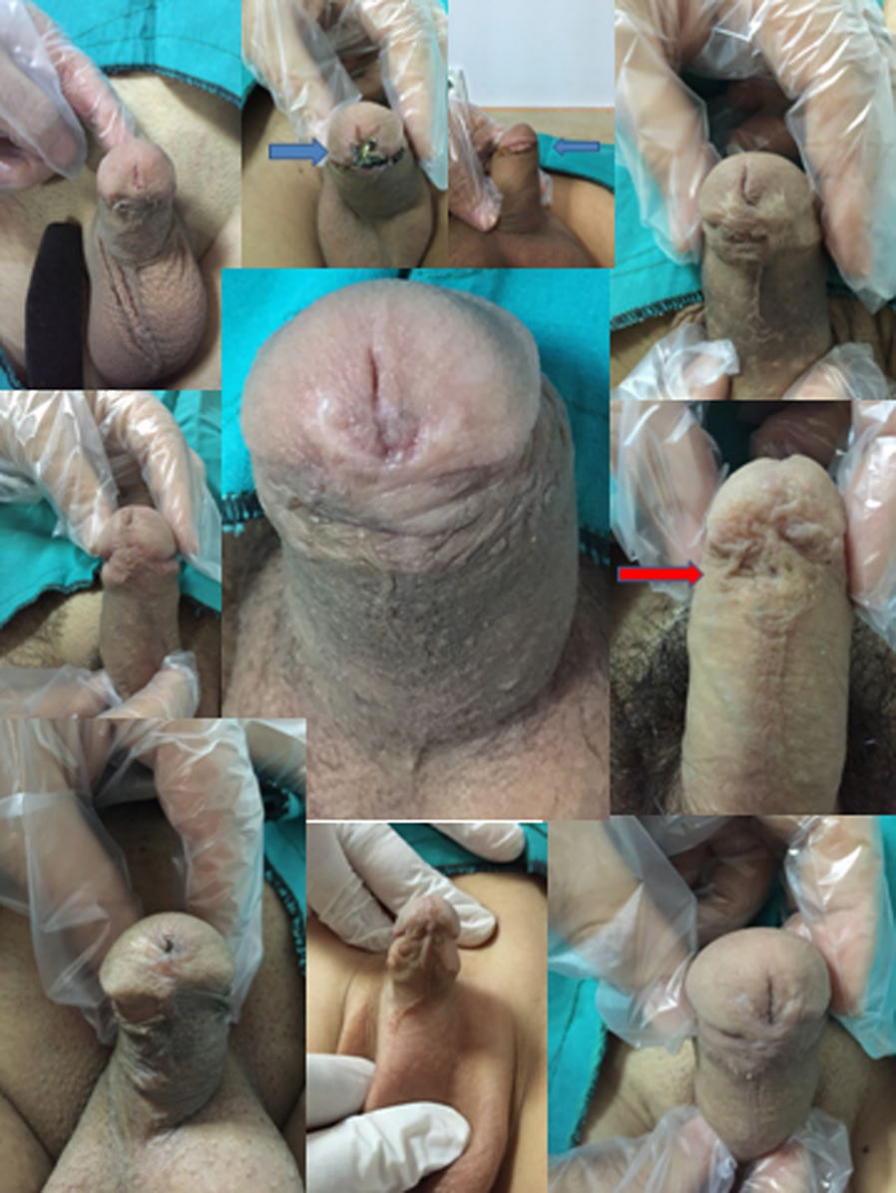
Images of some patients in postoperative different time

Perioperative outcomes, postoperative complications, and uroflowmetry findings are given in Table 2. Perioperative outcomes such as operation time, duration of urethral catheterization, and duration of postoperative hospital stay were similar in children and adult patients (*p* > 0.05). Regarding postoperative complications, urethrocutaneous fistulae developed in three (5.2 %) children, who fully recovered after fistula repair. Urethrocutaneous fistulae also developed in two (10.5 %) adults. Fistula repair was performed in one of these patients, and the patient fully recovered. The other patient has been scheduled for fistula repair. Glans dehiscence developed in one (5.3 %) adult; secondary TIP urethroplasty was performed in this patient, however, urethrocutaneous fistulae developed after this session, which were successfully repaired during a third surgery (Fig. 2).

**Table 2:** Perioperative and postoperative outcomes and complications

	Children	Adults	*p* value
Perioperative outcomes			
Operation time, min			
Mean ± SD	34.40 ± 8.938	34.21 ± 6.511	0.347
Median (min–max)	30.00 (25–60)	35.00 (25–50)	
Duration of urethral catheterization, h			
Mean ± SD	40.90 ± 7.878	36.89 ± 8.089	0.139
Median (min–max)	48.00 (24–48)	36.00 (26–52)	
Duration of hospital stay postoperatively, h			
Mean ± SD	43.31 ± 7.762	40.0 ± 7.824	0.181
Median (min–max)	48.00 (26–54)	38.00 (30–55)	
Postoperative complications			
Glans dehiscence, n (%)	0	1 (5.3)	0.237
Urethrocutaneous fistula, n (%)	3 (5.2)	2 (10.5)	0.587
Meatal stenosis, n (%)	1 (1.7)	0	1.000
Urinary tract infection, n (%)	2 (3.4)	0	1.000
Uroflowmetry findings			
Peak flow rate (mL/s)			
Mean ± SD	17.1 ± 2.998	20.42 ± 3.322	<0.0001
Median (min–max)	16.00 (14–28)	20.00 (16–29)	

*SD* standard deviation

The uroflowmetry peak flow rate (Q_max_) was measured; the mean was 17 mL/s (min–max 14–28) in the pediatric patients and 20 mL/s (min–max 16–29) in the adults. The flow pattern of all patients was normally shaped during uroflowmetry measurements.

No adult patients reported sexual dysfunction or disability after surgery. In addition, a patient aged 54 years with mid-penile hypospadias whose main issue was infertility fathered a child 1 year postoperatively.

There was no statistically significant difference between pediatric and adult patients in terms of meatus location on the penis and complications, such as urethrocutaneous fistula, glans dehiscence, urinary tract infection, and meatal stenosis (*p* > 0.05).

## Discussion

Many studies in the literature focus on pediatric patients who undergo primary hypospadias repair with TIP urethroplasty and modifications thereof (Tavakkoli Tabassi and Mohammadi 2010; Karakus et al. 2014; Acimi 2011). To our knowledge, there is only one publication related to the comparative success rates of TIP urethroplasty in adult and pediatric patients. In that study, Snodgrass et al. published the results of six adult patients who underwent primary repair with TIP urethroplasty for distal hypospadias (Snodgrass et al. 2014). Urethroplasty complications were not observed in these patients. The authors stated that there was no difference in the urethral complication rate in adults and children.

In a study somewhat similar to the present study, Hensle reported complications in three (37 %) of eight patients who underwent a primary repair (Hensle 2013). However, other hypospadias repair techniques were used in Hensle’s study, including various flaps and 1-stage tubed grafts. Hensle stated that these results clearly differed from those achieved in childhood repairs.

In addition, to our knowledge, three studies in the literature included only adult patients. Sharma published a series of 13 patients aged 18–26 years (Sharma 2005). A urethrocutaneous fistula developed in one patient in this series; no other complications were reported. Hatipoglu et al. performed a study on 27 men who were treated with primary distal (n = 19) or mid-shaft TIP urethroplasty (Hatipoglu et al. 2013). Complications developed in 5 (19 %) men, including 2 fistulae and 3 meatal stenoses. Adayener and Akyol described 12 men with distal hypospadias who underwent TIP urethroplasty (Adayener and Akyol 2006). A urethrocutaneous fistula developed in 1 of the 12 men (8 %) as a complication.

In this study, we shared the results of 77 consecutive patients who underwent primary hypospadias repair with TIP urethroplasty. Nineteen of these patients were adults. Our series showed that the success rate of adult hypospadias repair with TIP urethroplasty is good and equal to the success rate seen in boys who undergo the same surgery.

In our routine practice, we follow several basic steps that may differ from other surgeons who perform hypospadias repair. First, we only use TIP urethroplasty in distal hypospadias repair. We use 3/0 or 4/0 polyglactin sutures in adults and 4/0 or 5/0 polyglactin sutures in children. We prefer 8 or 10 F latex Foley catheters in children and 14 F latex Foley catheters in adults. We remove the catheter two or three days postoperatively, because we believe that early catheter removal reduces cuff formation (Polat et al. 2015).

There are two potential limitations of our study. First, only the results of patients who underwent primary repair for distal or mid-shaft hypospadias were analyzed. Second, the mean follow-up was 16 months; additional complications may be diagnosed after a longer follow-up.

## Conclusion

To our knowledge, this is the second study in the literature to compare hypospadias repair in children and adults. TIP urethroplasty for primary hypospadias repair was successful in both boys and adults, with a low complication rate. The success rates of TIP urethroplasty were the same in adults and children.

## Abbreviation

TIP urethroplasty: tubularized-incised plate urethroplasty
